# Observation of high nonlinearity in Bi doped Bi_x_In_35-x_Se_65_ thin films with annealing

**DOI:** 10.1038/s41598-021-01134-4

**Published:** 2021-11-02

**Authors:** P. Priyadarshini, Subhashree Das, D. Alagarasan, R. Ganesan, S. Varadharajaperumal, Ramakanta Naik

**Affiliations:** 1 Department of Engineering and Material Physics, ICT-IOC, Bhubaneswar, 751013 India; 2 Department of Physics, Indian Institute of Science, Bangalore, 560012 India; 3 Centre for Nano Science and Engineering, Indian Institute of Science, Bangalore, 560012 India

**Keywords:** Optics and photonics, Physics

## Abstract

The present work demonstrates the impact of thermal annealing on the structural, linear, and non-linear optical characteristics of thermally evaporated Bi_x_In_35-x_Se_65_ (x = 0, 5, 10, 15 at%) thin films. The prominent crystalline phases have been developed for all annealed films at 450 °C whereas the films remain amorphous at 350 °C annealing. The XRD and Raman analysis showed the phase transformation of Bi-doped films and new Bi_2_Se_3_ phases developed upon annealing at 450 °C. The phase transformation induced change increased the linear and nonlinear properties with great extent as seen from the UV–visible optical studies. The direct and indirect optical bandgaps decreased with annealing temperature and also with Bi % content due to the formation of surface dangling bonds near the crystallite sites. The static linear refractive index and high-frequency dielectric constants were increased with annealing. The third-order non-linear susceptibility and non-linear refractive index were found to be greatly influenced by annealing temperature and increased with bismuth content. The FESEM micrographs also showed the phase transformation and EDX analysis showed the composition. The results obtained from the materials showed the potentiality to be useful for photovoltaic and optoelectronic applications.

## Introduction

The amorphous chalcogenide materials have been intensively investigated because of their promising optoelectronic properties and hence possible applications in optical fibers, memory devices^1^, solar cells^2^, optical telecommunication^3^, and sensors^4^. The high non-linear refractive index of these materials makes them to play a significant role in applications such as supercontinuum, all-optical switching, and wavelength conversion^5,6^. With the increasing concerns and demands nowadays, improvements are needed to achieve the most resonant characteristics for device applications. So many studies have concentrated on the effect of external energy input such as doping of foreign elements^7^, thermal annealing^8^, laser irradiation^9^, ion irradiation^10^, etc. on the properties of the films. Among these processes, thermal annealing method is well known for the reduction in the structural defects and increment in the crystallite size that is responsible for the change in their structural and optical characteristics^11^. The influence of heating treatment on different properties has been studied by various researchers. Thermal annealing induces crystallization accompanied by the change in band gap and activation energy of Ga_15_Se_77_In_8_ thin film was studied by Al-Agel et al.^12^. The crystallized GeSe_2_ and Bi_2_Se_3_ phases with monoclinic and hexagonal crystal structures have been observed upon annealing Bi_5_Ge_40_Se_55_ thin films at different temperatures which results in the decrease in bandgap and changes in dielectric properties^13^. The annealing induced large non-linear optical changes in Ge_20_Se_65_S_15_ thin films suggests for use in low-power devices like optical computers, ultrafast switches, and pulsed lasers^14^.

In this regard, the In–Se (III-VI) system is considered as an archetypical semiconducting chalcogenide that takes an important place in applications such as electrical switching, nonlinear optics, diodes, photodetectors^15,16^. Several studies based on the additives in In–Se chalcogenide systems have grabbed much attention due to changes in their optoelectrical properties which may be seen as new optimistic applications of multicomponent chalcogenide systems^17–19^. Among them, bismuth as a dopant showed significant influence on the host InSe material by several modifications such as phase transformation, carrier type reversal, and change in optoelectrical properties which supports the understanding of the fundamental mechanisms inside the system^20^.

Previously, we have studied the doping effect of bismuth into Bi_x_In_35-x_Se_65_ thin films prepared by thermal evaporation technique which showed the presence of crystallinity behavior in 7% and 15% Bi-doped films with significant modification in linear and non-linear optical characteristics^21^. However, in the present report, we have focused on the analysis of the optical and structural properties of thermally annealed Bi_x_In_35-x_Se_65_ chalcogenide thin films. The glass transition temperature (T_g_) plays an important role in the characterization of glassy materials that represents the temperature above which an amorphous matrix attains various structural configurations. The T_g_ influenced different parameters such as average coordination number, enthalpy of atomization, mean bond energy, and bandgap^22^. Therefore, the annealing temperatures in glassy materials were taken by considering T_g_ value. In this regard, we have annealed the film with above T_g_ (at 350 °C and 450 °C) as our case study to investigate the influence of annealing temperature on different properties. The T_g_ for the Bi–In–Se system with low In% content were found to be 324 K (51 °C)^23^. The annealing effect on the Se_85_In_15-x_Sb_x_ films at temperatures above T_g_ showed improved linear optical properties of the host matrix which is due to the structural rearrangement that occurred from amorphous to crystalline transformation^18^. Furthermore, the appearance of binary Bi_2_Se_3_ and BiSe_2_ phases with the reduction in the optical bandgap and activation energy were observed due to annealing below T_g_ (~ 429 K) in Sn_10_Sb_20-x_Bi_x_Se_70_ (0 ≤ x ≤ 8) films that improved the device performance^24^. The increase in annealing temperature not only increases the crystallinity growth but also showed an increase in the absorption capability which causes blue shift of the optical bandgap^25^. Similarly, annealing of Bi_x_In_25-x_Se_75_ films at 440 K (167 °C) showed an appearance of crystallized Bi_2_Se_3_, Se, In_2_Se_3_ phases at x = 7 at % that induces an increase in optical bandgap which is explained by the thermal relaxation of the vapor quenched state and density of defect states^20^. However, the annealing-induced studies on the Bi–In–Se system were restricted up to 7% Bi doping. The annealing induced changes in higher Bi% and the changes in nonlinear parameters such as non-linear refractive index and third-order non-linear susceptibility with annealing is the prime aim of the present work.

The objective of the present study is to investigate the annealing induced effects on the linear and nonlinear optical parameters such as absorption coefficient, extinction coefficient, optical band gap, static refractive index, third-order optical susceptibility, nonlinear refractive index of thermally evaporated Bi_x_In_35-x_Se_65_ (x = 0, 5, 10, 15 at %) thin films at 350 °C and 450 °C annealing temperatures. The elemental composition and morphological identifications were done through Energy dispersive X-ray analysis (EDX) attached with the Field emission scanning electron microscope (FESEM). The annealing-induced structural modifications and phase transformation were investigated by X-ray diffraction method (XRD) and Raman spectra analysis. The optical constants have been estimated from the transmittance data obtained by UV–Vis spectrophotometer over the range 600–1100 nm wavelength.

## Experimental details

Bulk Bi_x_In_35-x_Se_65_ (x = 0, 5, 10, 15) samples were prepared from the stoichiometric mixture of high purity (99.999% Sigma Aldrich) Bi, In, and Se by conventional melt quenching process. The detailed procedure for the preparation of bulk and thin film samples are reported in our earlier study^21^. The glass slides were used as substrate for film deposition and were cleaned by dipping into hydrogen peroxide (H_2_O_2_), subsequently treated in trichloro ethylene, acetone, and methanol in an ultrasonic bath. The prepared thin films were annealed at 350 °C and 450 °C both at above T_g_ for 2 h under pressure 10^–3^ Torr, and the temperature gradient during the annealing process was ~ 5 to 6 °C/h.

The amorphous state and crystalline structure were studied by using XRD (Brucker D8 Advanced) with Cu K_α_ line (λ = 1.54 Å). The scanning range was 20°–60° at a step size of 0.02°/s with grazing angle 1° and scan speed 1°/min. The verification of the concentration, surface morphology, and elemental analysis of the annealed films was examined by EDX technique linked with FESEM unit (Carl Zeiss Ultra 55). The system was operated with accelerating voltage of 20 kV with emission current 40 mA for 1 cm^2^ sample size at 3–4 positions under high vacuum conditions (2 × 10^–7^ Torr). The Raman spectra (LabRAM HR system) for the annealed films were recorded by employing argon laser (514.5 nm) source over the range 50–40 °C m^−1^ through backscattering mode of CCD detector. The optical transmission data of the annealed films were recorded by using UV–Vis spectrophotometer (Bruker Optics (IFS66v/S)) over 600–1100 nm range. The optical parameters such as absorption constants, optical density, skin depth, extinction coefficient, direct and indirect bandgap, Tauc parameters for the studied films were estimated and discussed by using suitable empirical relations and models. The linear static refractive index (n_0_), high-frequency dielectric constant (ε_∞_), and non-linear parameters such as third-order non-linear susceptibility (χ^(3)^), non-linear refractive index (n_2_) were calculated from Miller’s rule and Dimirov-Sakka empirical relation.

## Results and discussions

### Structural analysis

#### X-ray diffraction study

**Figure 1:**
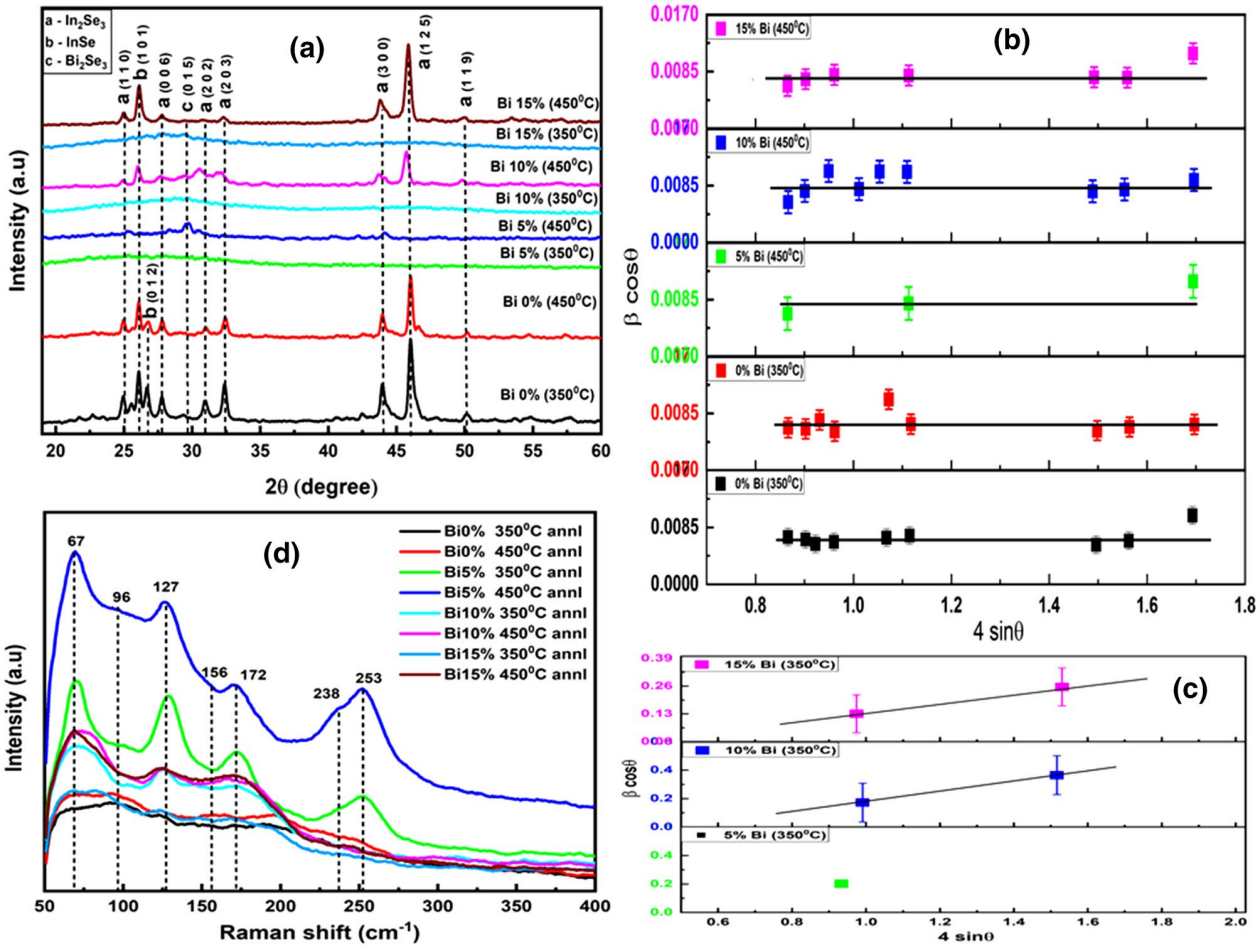
(**a**) XRD patterns, (**b**) Williamson-Hull plot for crystalline and (**c**) amorphous peaks, (**d**) Raman spectra of the annealed Bi_x_In_35-x_Se_65_ thin films.

The XRD spectrum of the annealed Bi_x_In_35-x_Se_65_ thin films is shown in Fig. 1a which confirms the amorphous nature for 350 °C annealed 5%, 10%, and 15% Bi-doped films. However, the 450 °C annealed films showed polycrystalline nature. The hexagonal phase of γ-In_2_Se_3_ has been represented by diffraction peaks such as 25.00° (1 1 0), 27.77° (0 0 6), 30.95° (2 0 2), 43.92° (3 0 0), (ICSD card: 00-023-0294) and 45.98° (1 2 5), 32.35° (2 0 3), 50.10° (1 1 9) (ICSD card: 01–071-0250) respectively. The observed diffraction peaks support the result obtained various workers^26–30^. The other peaks at 26.06° (1 0 1), 26.65° (0 1 2) corresponds to rhombohedral phase of InSe (ICSD card: 00–029-0676) have been indexed in the figure. The subsequent annealing after the introduction of bismuth in In–Se alloys resulted in an additional diffraction peak at 29.29° which represents the rhombohedral Bi_2_Se_3_ (0 1 5) (ICSD card: 00-033-0214) respectively. The different phases obtained in this study are matching with the reported data^31^. The XRD analysis of these annealed Bi-doped (5%,10%, and 15% Bi) samples enables us to ensure the phase transformation from amorphous to crystalline phase while annealing from 350 to 450 °C that supports several studies^32,33^. The amorphous nature of 350 °C Bi-doped annealed films signifies no structural transformation inside the films annealed at 350 °C. This behavior may be due to the incorporation of high-density bismuth atoms that increases the disorder and density inside the system^34^. The appearance of polycrystallinity in all the 450 °C annealed films induces crystallization in semiconductor chalcogenide films. In the case of In_35_Se_65_ films, the increase in annealing temperature from 350 to 450 °C decreased the intensity and, sharpness of the crystalline peaks indicating to decrease in crystallinity^35^.

Several structural parameters were estimated by using the well-known Scherrer and the Williamson-Hall equations of the sample. Based on the Scherrer equation, the crystallite size D (in nm) is inversely dependent on peak width β (full-width half maxima) as follows^36^,

1 $$ Crystallite\;size \left( D \right) = \frac{0.9\lambda }{{\beta \cos \theta }} $$

where λ is the wavelength of Cu K_α_-line (1.54 Å), β is the full-width half maxima (FWHM) in radians and θ is the Bragg’s angle. The values of average crystallite size at different temperatures for different Bi concentrations are tabulated in Table 1. The crystallite size increased with the annealing temperature which is due to the increase in the grain size as a result of an increase in the mobility and migration of atoms. During the thermal annealing process, the atoms receive enough energy that helps them migrate to relative equilibrium positions. This causes an enhancement of the grain growth and decreases of lattice dislocation in the films^36^ as a result of which there is increased formation of lattice grains and lattice imperfections.

**Table 1:** Calculated lattice parameters of annealed Bi_x_In_35-x_Se_65_ thin films.

Samples	Debye–Scherrer method			Williamson Hull method		
	Average crystallite size (nm)	N_c_ (nm^−2^)	Dislocation density (δ) nm^−2^	D (nm)	ε × 10^–4^	Dislocation density (δ) nm^−2^
Bi 0% 350 °C annl	20.31	0.0954	0.002424	19.09	6.11	0.002744
Bi 0% 450 °C annl	20.29	0.0957	0.002429	18.19	5.54	0.003022
Bi 5% 450 °C annl	16.00	0.1953	0.003906	18.03	7.41	0.003076
Bi 10% 450 °C annl	16.37	0.1823	0.003731	16.68	4.98	0.003594
Bi 15% 450 °C annl	17.74	0.1432	0.003177	20.81	7.17	0.002309

The other microstructural parameters such as lattice strain (ε), dislocation density (δ), the number of crystallites per unit surface area (N_c_) of the films were calculated using the following relations^37^,

2 $$ Lattice\;strain \left( \varepsilon \right) = \frac{\beta \cot \theta }{4}, $$

3 $$ Dislocation\;density \left( \delta \right) = \frac{1}{{D^{2} }},\quad {\text{and}}\quad N_{c} = \frac{d}{{D^{3} }} $$

The obtained values for dislocation density and N_c_ from average crystallite size were tabulated in Table 1. It is seen that bismuth incorporated annealed films showed higher dislocation, strain, and lower crystallite size with respect to In_35_Se_65_ film, which is due to an increase in the densification with doping that leads to the formation of smaller grains. Overall, decreasing behavior in the dislocation density inside the crystal is due to the refinement of crystallite size and annihilation of dislocation inside the films which indicates an increase in the crystallinity of the material^38^. The N_c_ decreased with annealing temperature may be due to the change in structure or formation of smaller crystallites^39^.

The simultaneous effect on the lattice strain and crystallite size on the peak broadening, the Williamson-Hall relation was used which is given by^40,41^,

4 $$ \frac{\beta \cos \theta }{\lambda } = \frac{k}{D} + \frac{4\varepsilon \sin \theta }{\lambda } $$

5 $$ \Rightarrow \beta \cos \theta = \frac{k\lambda }{D} + 4\varepsilon \sin \theta $$

The first term on the right-hand side of the equation demonstrates the Scherrer equation, which signifies the effect of crystallite size. Whereas, the second part shows the change in microstrain associated with nanoparticles on the broadened peak which is known as the Stokes and Wilson expression. Generally, the width β in a diffraction peak gets influenced by the change in instrumental factors, change in strains, crystallite size, and presence of crystal defects. When the peak broadening shows independence over 1/D, an enhancement in 1/D values increased the strain broadening. In this case, the size and strain of the crystallites are evaluated simultaneously. Therefore, the W–H plots were presented in Fig. 1b and c by taking (β cosθ) in the Y-axis and (4sinθ) in the X-axis, where the crystallite size (D) = kλ/(y-intercept), and the slope gives the strain values in Fig. 1b tabulated in the Table 1. The estimated values were very different from the value obtained in the Scherrer equation. In general, the W–H plot shows both positive and negative slopes, where positive values correspond to tensile strain and negative values to compressive strain. The estimated strain values for 350 °C annealed films in Fig. 1c are 0.225 (15% Bi), 0.366 (10% Bi), and corresponding crystallite size are 1.554 (15% Bi), 0.723 (10% Bi) respectively*.* In our case, all the values were positive slopes which appear as tensile strain may be due to contact and coherency stress, grain boundary, stacking faults^41^. This results in the shifting of peak position was observed from Fig. 1a.

#### Raman analysis

Raman spectroscopic analysis is a useful chemical analysis technique that provides useful information on molecular interactions, crystallinity, crystal phase, and chemical structure. The significant information on the structural rearrangements and phase transitions of the samples due to annealing are obtained from spectral analysis of vibrational levels. The Raman spectra of the 350 °C and 450 °C annealed Bi_x_In_35-x_Se_65_ thin films are shown in Fig. 1d that showed different peaks in between 50 and 40 °C m^−1^ range. The In_35_Se_65_ spectrum contains peaks at 96 cm^−1^, 156 cm^−1^, 238 cm^−1^, among which the former two peaks (96 cm^−1^, 156 cm^−1^) are related to the In–Se phase^41,43^. The weaker broad peak at 238 cm^−1^ is attributed to the homopolar Se–Se vibrational chain^30^ that disappeared after Bi doping except for 450 °C annealed 5% Bi-doped film. New additional peaks at 67 cm^−1^, 127 cm^−1^, 172 cm^−1^, and 253 cm^−1^ arise due to Bi doping and subsequent annealing of Bi–In–Se films. The peak at 67 cm^−1^ corresponds to the E_g_^1^ mode of the Bi–Bi vibrational bond^44^. Two prominent peaks at 127 cm^−1^, 172 cm^−1^ are assigned to E_g_^2^ and A_1g_^2^ mode of Bi_2_Se_3_ band^45^, and the broad sharp Raman vibrational peak at 253 cm^−1^ is due to the presence of Se_n_ rings^43,46^. This broad peak shifts slightly towards higher wave number which shows the annealing induced structural modification due to the evolution of crystalline phases. Among all the annealed films, 5% Bi-doped annealed film peak showed the maximum intensity that indicates the greater structural modifications inside the matrix and decreases simultaneously for higher Bi-doped films. The overall increases in Raman intensity of all the peaks and the slight shifting to high wavenumbers with an increase in the annealing temperature indicate significant structural change in the glass structure and the formation of micro crystallites^14^. Further, on increasing Bi concentration from 5 to 15%, Raman intensity decreases and broadening increases due to an increase in lattice defect concentration which is confirmed from XRD analysis^47^.

### Morphological analysis

#### EDX analysis

**Figure 2:**
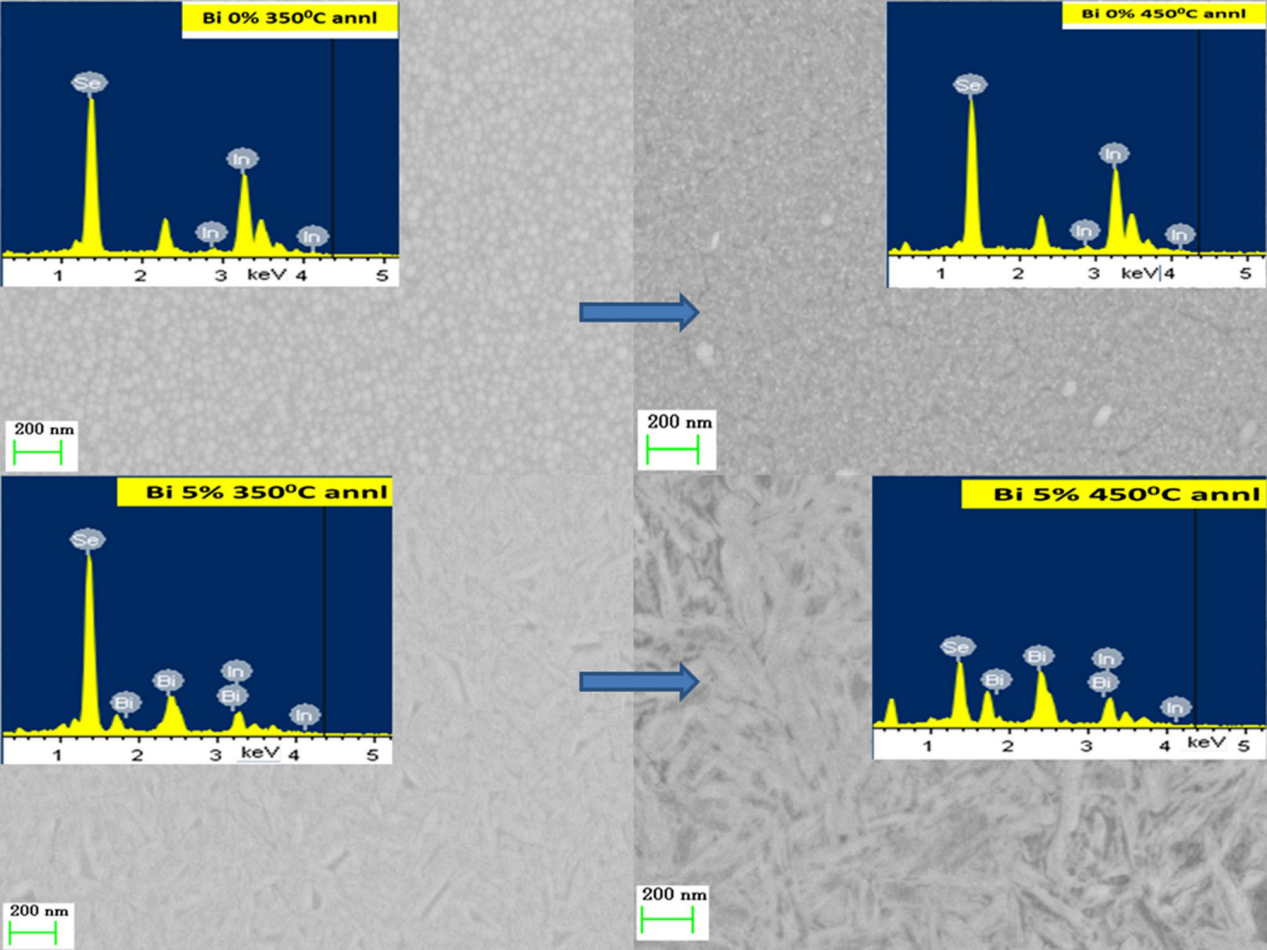
FESEM images of 350 °C and 450 °C annealed In_35_Se_65_ and Bi_5_In_30_Se_65_ thin films.

**Figure 3:**
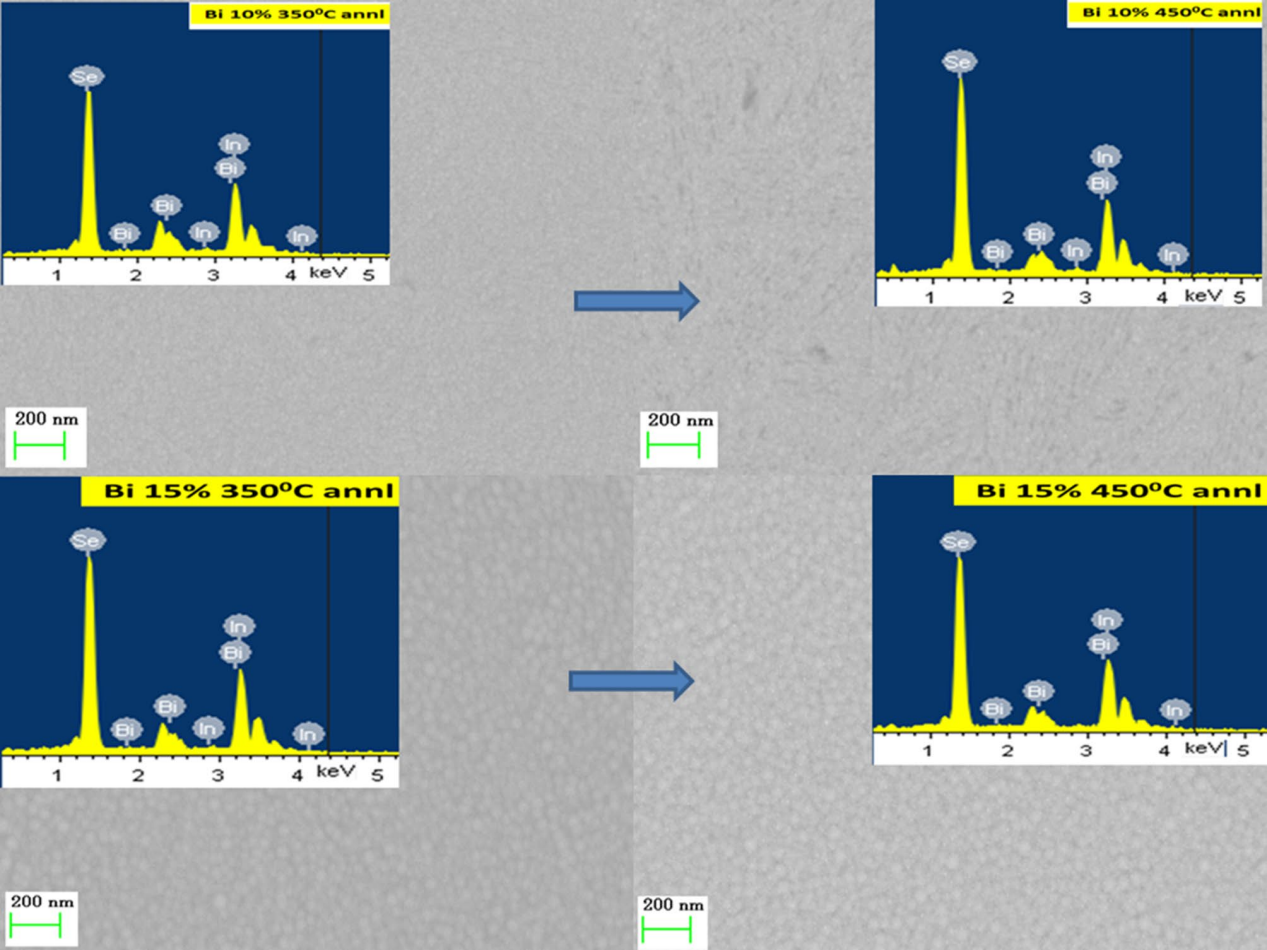
FESEM images of 350 °C and 450 °C annealed Bi_10_In_25_Se_65_ and Bi_15_In_20_Se_65_ thin films.

The EDX pictures are shown in Figs. 2 and 3 that represent the presence of the constituent elements in the 350 °C and 450 °C annealed Bi_x_In_35-x_Se_65_ thin films. The In_35_Se_65_ annealed film as shown in Fig. 2 showed the presence of only In and Se whereas the other annealed Bi_5_In_30_Se_65_ films showed the evidence of bismuth content through Bi-peaks along with In and Se peaks. Similarly, different peaks correspond to constituent elements present in the Bi_10_In_25_Se_65_ and Bi_15_In_20_Se_65_ thin films annealed at 350 °C and 450 °C can be seen in Fig. 3. The composition of the different annealed films is shown in Table 2 which shows nearly equal concentration as that of the calculated values. The error in composition with annealing is within 3%. The peak at 2.25 keV in the In_35_Se_65_ annealed films may be due to indium concentration which got unseen due to the presence of the Bi peak in Bi-doped films^48^. The suppression of selenium peak in the Bi 5% doped 450 °C annealed film occurred along with slight increase with Bi peak intensities. This kind of behaviour has also seen in reference^49^ which is due to an increase in the annealing temperature. This suppression behaviour has not observed in other films which may be due to the increase in the Bi concentration in the samples.

**Table 2:** Composition of different annealed Bi_x_In_35-x_Se_65_ (x = 0, 5, 10 and 15) thin films.

Sample	Bi 0%			Bi 5%			Bi 10%			Bi 15%		
Temp		350 °C	450 °C		350 °C	450 °C		350 °C	450 °C		350 °C	450 °C
Element	Cal (At.%)	Obs (At.%)	Obs (At.%)	Cal (At.%)	Obs (At.%)	Obs (At.%)	Cal (At.%	Obs (At.%)	Obs (At.%)	Cal (At.%)	Obs (At.%)	Obs (At.%)
In	35	34.52	34.61	30	29.35	29.39	25	24.29	24.24	20	19.39	19.46
Se	65	65.48	65.39	65	65.81	65.70	65	66.18	66.12	65	65.85	65.72
Bi	0	0	0	05	04.84	04.91	10	09.53	09.64	15	14.76	14.82
Total	100	100	100	100	100	100	100	100	100	100	100	100

#### FESEM analysis

**Figure 4:**
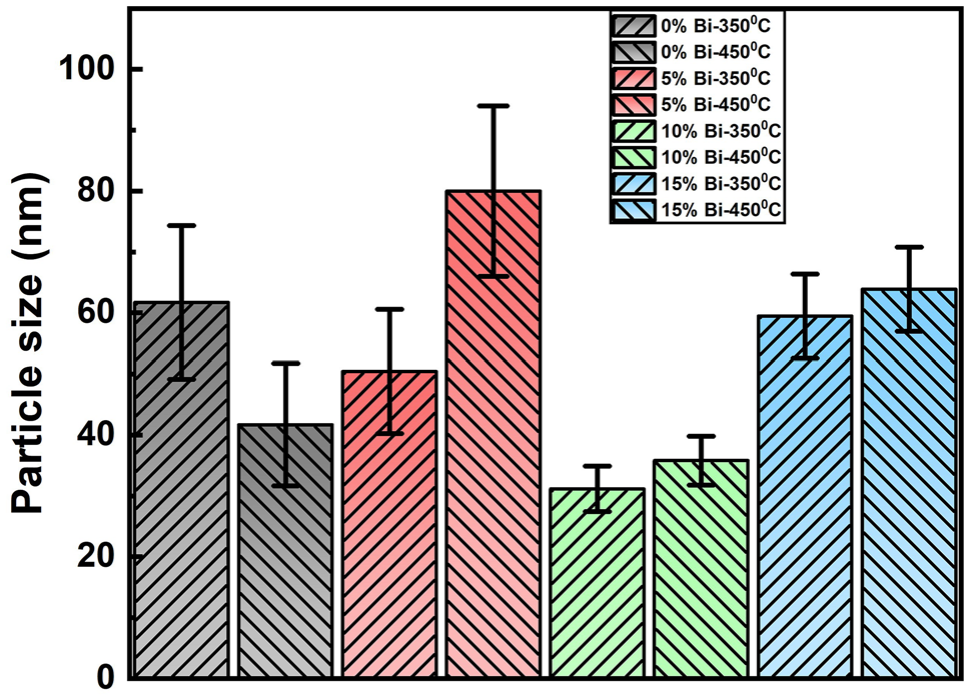
The histogram of the particle size distribution in the annealed films.

The FESEM images at 200 nm scale for 350 °C and 450 °C annealed Bi_x_In_35-x_Se_65_ films are shown in Figs. 2 and 3 respectively. The images show the homogeneous and smooth nature of the annealed films. Annealing at 450 °C results in an increase in particle density which can be observed from both the figures as compared with the 350 °C annealed films. We have calculated the particle size for each sample by using ImageJ software (version -Java 1.8.0_172) and done the statistical analysis which is shown in Fig. 4. It can be observed that the particle size increased with increase in annealing temperature i.e., from 350 to 450 °C. The overall particle size decreased considerably with higher bismuth concentration which can be seen from the histogram distribution. The Bi 5% annealed film at both 350 °C and 450 °C shows the particles in the form of nanorod like structures. Similarly, the Bi 10% annealed film at both the temperatures shows particles with fragmentation form.

### Optical data analysis

#### Linear optical parameters

##### Transmittance (T) and absorption coefficient (α)

**Figure 5:**
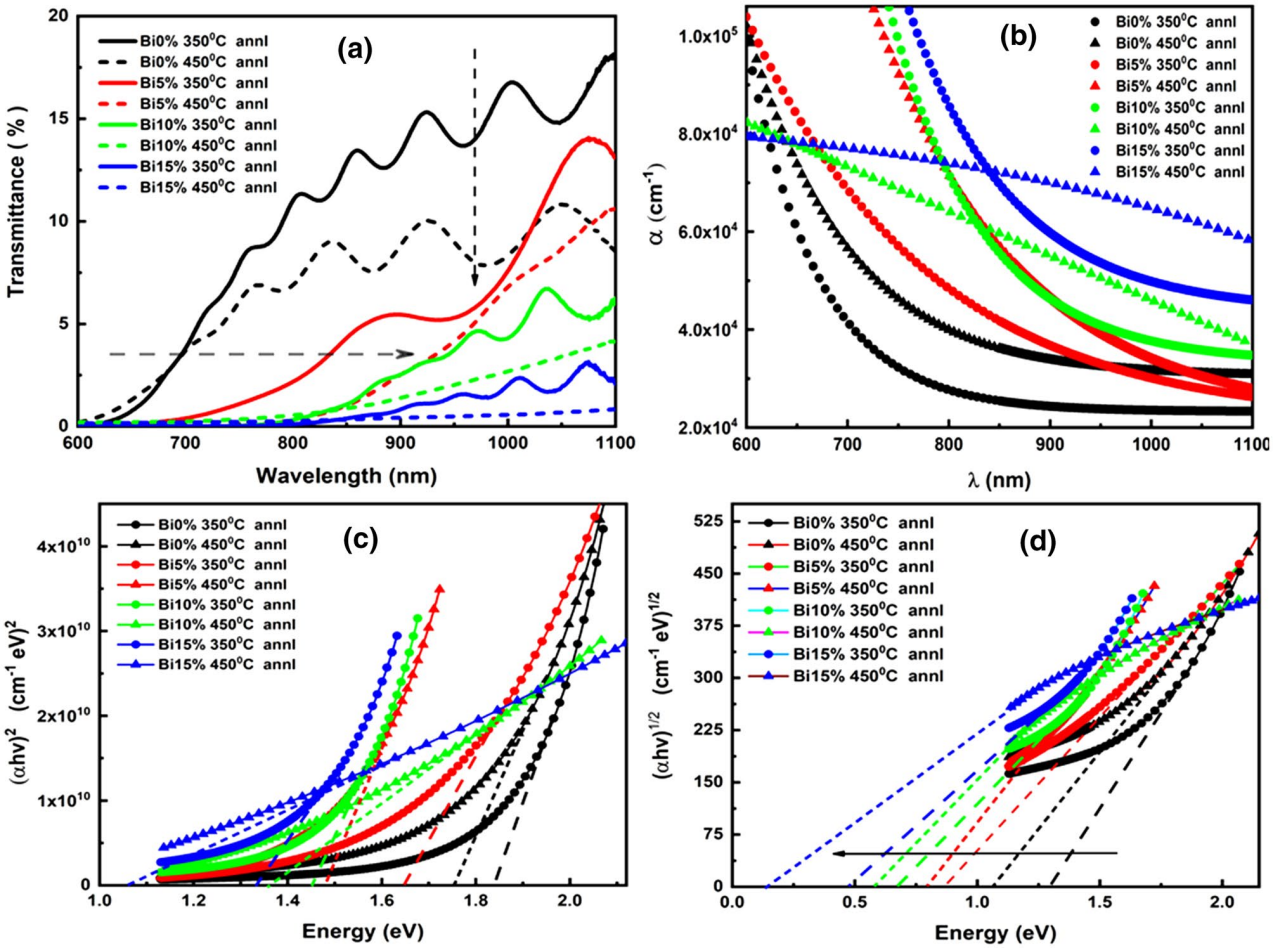
(**a**) Transmittance spectra of annealed the Bi_x_In_35-x_Se_65_ films (**b**) Absorption coefficient (α) vs wavelength spectra for the annealed Bi_x_In_35-x_Se_65_ films (**c**) Direct optical bandgap calculation for the annealed Bi_x_In_35-x_Se_65_ films (**d**) Indirect bandgap calculation for the annealed Bi_x_In_35-x_Se_65_ films.

The UV–visible transmission study provides valuable information regarding the response of materials towards light. Electromagnetic waves with weak or moderate intensity interact with the material, leads to an induced polarization, and are affected linearly by the electric field. These linear responses can be explained through reflection, transmission, absorption, or scattering. So, these linear optical characteristics play an important role in the application perspective^50^. The transmission spectra of annealed Bi_x_In_35-x_Se_65_ (x = 0, 5, 10, 15 at %) films for the wavelength interval of 600–1100 nm are presented in Fig. 5a. The transmission % decreased with an increase in both annealing temperature as well as dopant concentration as reported for other studies also^51,52^. The vertical arrow signifies the decrease in transmittance of the samples whereas the horizontal arrow signifies the shifting of absorption edge towards higher wavelength. It can be clearly observed that annealing at 350 °C films showed better transmittance than at 450 °C annealed films. The decreased transmittance with annealing temperature is accompanied by the structural transformation of the film as a result of the growth of crystallites in the material^53^.

The absorption edge shifted towards higher wavelength with increasing annealing temperature and Bi concentration. This red shift of absorption edge is due to an enhancement in grain boundary defects created by the crystallites formed due to annealing which decreases the optical bandgap. The appearance of transmission fringes nearly at 700 nm beyond the visible wavelength of light in some films (350 °C annealed films) provides good evidence on the interference phenomenon of light with the material medium that indicates the homogeneity and smoothness throughout the film thickness^54^.

The optical absorption coefficient (α) is considered as an important parameter for the evaluation of the optical band gap and Tauc parameter of the thin films. The parameter ‘α’ provides information on the absorption of light energy during the electronic transition. The ‘α’ value was calculated from the measured values of transmittance (T) by using the following equation^55^;

6 $$ \alpha = \frac{1}{d}\ln \left( \frac{1}{T} \right) $$

where ‘d’ represents the thickness of the film (~ 800 nm). The absorption coefficient spectra for the 350 °C and 450 °C annealed Bi_x_In_35−x_Se_65_ thin films as a function of wavelength is shown in Fig. 5b. The obtained absorption coefficient is of the order of 10^4^–10^5^ cm^−1^ which showed good agreement with the results by various workers. It can be noticed that the absorption coefficient decreased with wavelength which satisfies the increase in the transmittance behavior at higher wavelength region. The absorption coefficient showed redshift on increase in the annealing temperature as well as with doping content. The 450 °C annealed 10% and 15% Bi-doped films showed different behavior than other films. This behavior can be explained by using the relationship between free carrier absorption in solids and the number of carriers. As the number of carriers increases during an increase in annealing which enhances the absorption capability significantly^56^. In-other way it can be explained through structural aspects of the modification through phase transition. The grain boundaries formed during phase transition indicate an increase in crystallinity and significantly affect the transmittance and absorbance behavior^57^. The high concentration of heavily dense bismuth increases the absorption significantly and correspondingly decreases the transmittance. Thus, in Fig. 5b, the 10% and 15% Bi-doped 450 °C annealed films showed a higher absorption value over the wavelength range. Such type of behavior has also been seen in the absorption coefficient (α) plot of N. M. Shah et al.^58^ work for high-temperature annealing.

##### Optical band gap (E_g_) and Tauc parameter (B^1/2^)

The optical band gap (E_g_) of the studied films from the high absorption region was calculated by using Tauc relation^59^;

7 $$ \alpha hv = B\left( {hv - E_{g} } \right)^{p} $$

where ‘B’ is known as the Tauc parameter which represents the degree of disorder in the materials and depends on the transition probability, ‘ν’ is the frequency of the incident beam, E_g_ is the optical band gap, p (exponent) is related to the nature of various electronic transitions. The value of ‘p’ has different values such as ½, 2, 3/2, and 3 depending on the different types of transition such as direct allowed, indirect allowed, direct forbidden, indirect forbidden respectively. According to Tauc, the amorphous materials showed indirect allowed bandgap whereas crystalline possesses direct allowed bandgap respectively. Since, here as a consequence of annealing induced phase transformation, both the amorphous (350 °C annealed 5%, 10%, and 15% Bi-doped films) as well as crystalline structure have been observed, thus we are concentrating on both direct and indirect allowed transition for the films. The optical band gap (E_g_) and Tauc parameters of different annealed samples were obtained from the slope and intercept obtained by plotting the dependence of (αhv)^1/p^ on photon energy(hv) and extrapolating the linear part of the curves to the energy axis for zero absorption coefficient. Figure 5c and d presents the variation of (αhv)^2^ with incident energy (hv) for directly allowed transitions and (αhv)^1/2^ with incident energy (hv) for indirect allowed transitions in annealed Bi_x_In_35-x_Se_65_ films. The horizontal arrow in the Fig. 5d represents the decrease in the indirect optical band gap values for the studied films. The calculated values of E_g_ and Tauc parameters for both direct and indirect transitions are shown in Table 3. The observed indirect bandgap values decreased as found in Table 3. The obtained values agree with those obtained by other investigations^20,25^. The direct bandgap values also decreased with annealing like indirect bandgap for the studied films. From Fig. 5c and d, it is observed that the optical band gap values decreased upon an increase in the annealing temperature from 350 to 450 °C which is ascribed to the annealing induced phase transformation in the evaporated films. Generally, annealing above the glass transition temperature (T_g_) causes crystallization inside the alloy along with the production of surface dangling bonds around the formed crystallites during the crystallization process. According to the ‘density of state model’ proposed by Mott and Davis^60^, an ideal amorphous solid shows phase transformation under heat treatment, and during the crystallization process, dangling bonds were produced around the crystallite surfaces. Further annealing causes the crystallite to break down into micro crystallites, thereby increasing the number of surface dangling bonds^61^. These bonds were responsible for the formation of defects in the polycrystalline solids. As the number of dangling bonds and defects increases, the concentration of localized states in the band structure gradually increases. Hence, an increase in the energy width of the localized state thereby reduces the bandgap^61,62^. Such type of behavior supports the results of various workers^63,64^. The subsequent increase in the bismuth content in the annealed films causes an increase in the density defect states by creating more localized states over the bandgap which consequently decreases the bandgap values^65^.

**Table 3:** Linear and nonlinear optical parameters of the annealed Bi_x_In_35-x_Se_65_ thin films.

Optical parameters	Bi 0%		Bi 5%		Bi 10%		Bi 15%	
	350 °C	450 °C	350 °C	450 °C	350 °C	450 °C	350 °C	450 °C
Indirect bandgap (E_g_^Ind^) eV	1.30	1.06	0.86	0.79	0.68	0.58	0.47	0.15
Tauc parameter (B^1/2^)^Ind^ cm^−1/2^ eV^−1/2^	560	446	382	432	370	364	313	261
Direct bandgap (E_g_^Dir^) eV	1.84	1.75	1.64	1.48	1.45	1.36	1.33	1.05
Tauc parameter (B^2^ × 10^10^)^Dir^ cm^−2^ eV^−2^	15.67	12.69	9.76	13.63	12.18	4.03	8.39	2.61
Static linear refractive index (n_0_^Ind^)	3.125	3.321	3.530	3.618	3.777	3.951	4.191	5.713
Static linear refractive index (n_0_^Dir^)	2.809	2.853	2.911	3.004	3.023	3.082	3.103	3.330
The dielectric constant of the lattice (ε_∞_^Ind^)	9.766	11.031	12.467	13.094	14.269	15.616	17.569	32.641
The dielectric constant of the lattice (ε_∞_^Dir^)	7.890	8.141	8.476	9.028	9.141	9.504	9.633	11.093
First order non-linear susceptibility (χ^(1)^) ^Ind^	0.698	0.798	0.913	0.962	1.056	1.163	1.319	2.519
First-order non-linear susceptibility (χ^(1)^) ^Dir^	0.548	0.568	0.595	0.639	0.648	0.677	0.687	0.803
Third-order non-linear susceptibility (χ^(3)^ × 10^–9^ esu)^Ind^	0.040	0.069	0.118	0.146	0.211	0.311	0.514	6.846
Third-order non-linear susceptibility(χ^(3)^ × 10^–11^ esu)^Dir^	1.540	1.777	2.134	2.837	3.001	3.573	3.795	7.088
Non-linear refractive index (n_2_ × 10^–9^ esu)^Ind^	0.486	0.784	1.260	1.522	2.112	2.972	4.629	45.15
Non-linear refractive index (n_2_ × 10^–10^ esu)^Dir^	2.065	2.346	2.762	3.558	3.740	4.367	4.607	8.019

The Tauc parameter (B^1/2^) is inversely proportional with the degree of disorder and is dependent on the nature of bonding which measures the disorderness. Here, the B^1/2^ value for Bi_x_In_35-x_Se_65_ films decreases upon annealing which is due to the increase in chemical disordering induced by the annealing process that increases the defect states and reduces bandgap. This decrease in optical band gap upon annealing showed the potentiality of these films to act as an absorber layer in various photovoltaic applications^66,67^.

##### Optical density (OD) and skin depth (δ)

**Figure 6:**
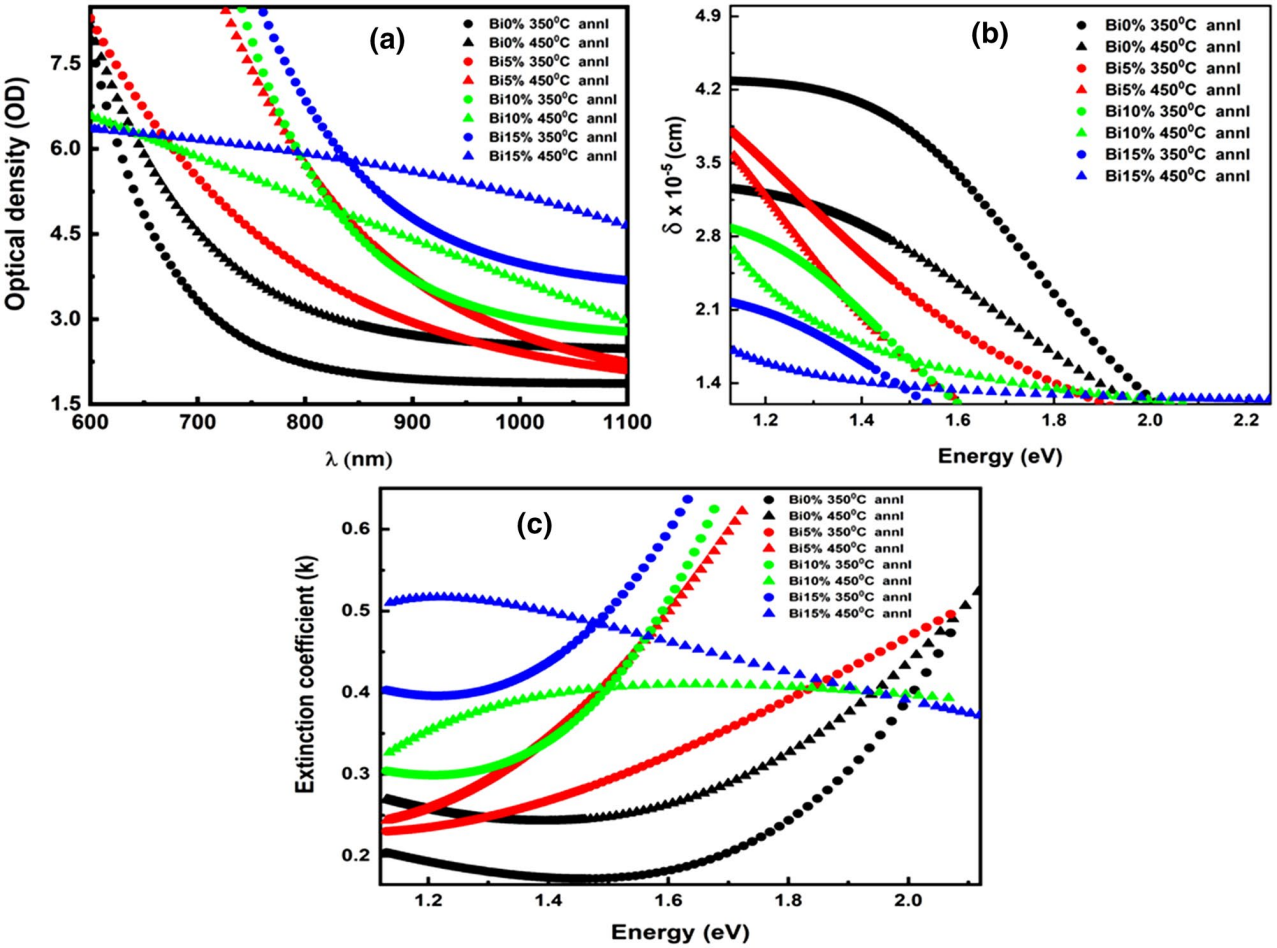
(**a**) Optical density, (**b**) skin depth, (**c**) extinction coefficient spectra for the annealed Bi_x_In_35-x_Se_65_ films.

The optical density (OD) or absorbance is related to the film thickness (d) and the concentration of absorbing material. The optical density of the presented annealed Bi_x_In_35-x_Se_65_ films can be estimated as^68^ **OD = α × d**. Figure 6a illustrates the variation of optical density (OD) with wavelength (λ) which shows the same variation as the absorption coefficient. The increasing trend of the OD values with respect to annealing temperature and with Bi doping concentration is due to high absorption coefficient values. This behavior clearly gives an indication of the increase in the absorption ability of the materials when exposed to incident radiation.

Skin depth (δ) represents the distance at which optical photon density becomes 1/e of the value at the surface after traveling the film thickness. The skin depth or penetration depth (δ) is related to the absorption coefficient (α) by the relation given by^32^, **δ = 1/α**. The dependency of skin depth on the incident photon energy (hv) is shown in Fig. 6b. It is clearly observed that the skin depth reduced to zero value for all films as the energy increases. The increase in annealing temperature in the studied films decreases the skin depth sequentially indicating loss in the transparency of the thin films.

##### Extinction coefficient (k), linear static refractive index (n_0_) and high frequency dielectric constant (ε_∞_)

The study of extinction coefficient (k) is extremely important to decide the usefulness of the prepared materials for application in photocatalytic, photochemical, photosensors, phase change materials, memory devices, etc. The extinction coefficient relates the extent of fraction of light losses due to scattering and absorption per unit distance of the medium that has been calculated by using absorption coefficient (α) and wavelength by following relation^53^,

8 $$ k = \frac{\alpha \lambda }{{4\pi }} $$

The variation of ‘k’ with the energy of incident radiation is shown in Fig. 6c that showed an incremental behavior with annealing temperature. This behavior is due to the thermal annealing process that creates several structural disorders and surface defects by producing localized states thereby increasing the absorption of light. The change in extinction coefficient immensely affects the non-linearity of the materials.

The linear static refractive index (n_o_) and high-frequency dielectric constant, ε_∞_ = n_0_^2^ of the annealed Bi_x_In_35-x_Se_65_ films were estimated by using the Dimirov and Sakka relation^69^;

9 $$ \frac{{n_{0}^{2} - 1}}{{n_{0}^{2} + 2}} = 1 - \sqrt {\frac{{E_{g} }}{20}} $$

where E_g_ corresponds to optical band gap estimated by using Tauc relation. The obtained values of static refractive index and high-frequency dielectric constant were tabulated in Table 3. From the obtained values, it is clearly observed that the refractive index increases with annealing temperature and also with Bi doping content. This increment is due to the amorphous-crystalline transition and lattice relaxation as a consequence of annealing^70^. This behavior also satisfied Moss’s rule i.e., E_g_n^4^ ~ constant^71^, which showed the optical band gap variation showed the opposite trend with respect to refractive index (n_o_). The dielectric constant also increased with an increase in annealing temperature.

#### Nonlinear optical parameter

##### Nonlinear susceptibility (χ^(3)^)

**Figure 7:**
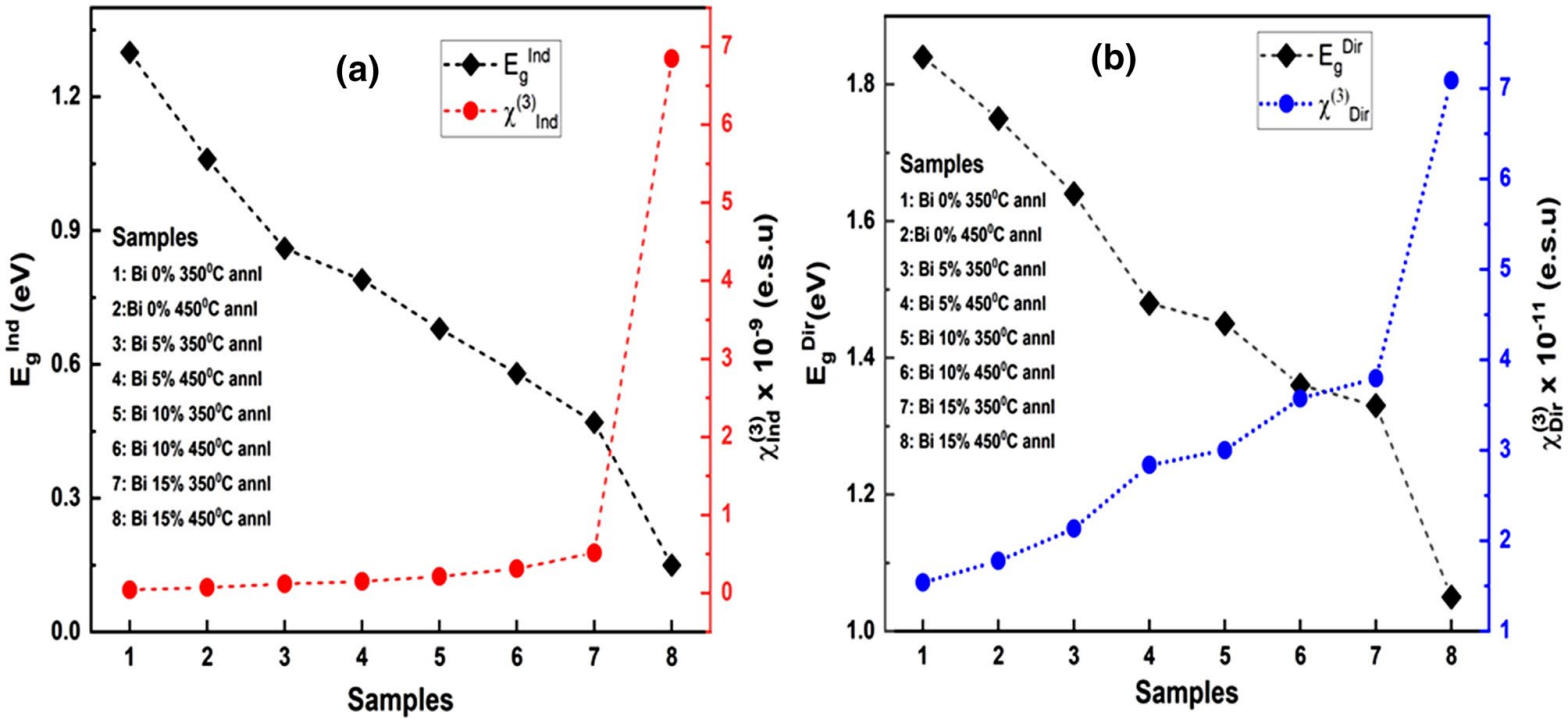
Variation of (**a**) E_g_^Ind^ and χ^(3)^, (**b**) E_g_^Dir^ and χ^(3)^ with annealed samples.

The nonlinearity of glassy materials depends on the electric field strength that is responsible for nonlinear effects in the system. Interaction in the nucleus due to the electronic polarization and also their impact on bond lengths is responsible for optical nonlinearities in the chalcogenide glasses^72^. Thus, the total electron polarizability(P) created due to such interaction can be presented as^73^;

10 $$ P = \chi^{\left( 1 \right)} E + P_{NL} ,\quad {\text{where}}\quad P_{NL} = \chi^{\left( 2 \right)} E^{2} + \chi^{\left( 3 \right)} E^{3} $$

where χ^(1)^ is the linear optical susceptibility, χ^(2)^ and χ^(3)^ are the second-order and third-order nonlinear susceptibility respectively. For centrosymmetric optically isolated glasses the second-order non-linear susceptibility is 0. Thus, the third-order nonlinear susceptibility is the dominant nonlinearity in all glassy materials that produced by excitation in the transparent frequency region below the bandgap E_g_. Therefore, according to Miller’s rule the χ^(1)^ and χ^(3)^ of annealed Bi_x_In_35-x_Se_65_ thin films can be obtained by following relation^74^,

11 $$ \chi^{\left( 1 \right)} = \frac{{n_{0}^{2} - 1^{^{\prime}} }}{4\pi }\quad {\text{and}}\quad \chi^{\left( 3 \right)} = A\left( {\chi^{\left( 1 \right)} } \right)^{4} = A\left( {\frac{{n_{0}^{2} - 1^{^{\prime}} }}{4\pi }} \right)^{4} $$

where n_0_ is the static refractive index for hv → 0 and A is a constant having value ~ 1.7 × 10^–10^ e.s.u. The obtained linear and non-linear susceptibility values for direct and indirect electronic transitions are tabulated in Table 3. It is observed that the third-order susceptibility χ^(3)^ value for both transitions increased with annealing temperature and Bi doping concentration. The monotonical increase of susceptibility for both transitions with decreasing bandgap is clearly shown in Fig. 7a and b. The increase in the susceptibility upon annealing is due to the change in the material structure as a result of phase transformation. In other words, the thermal annealing process allows an enhancement of homogenization and polymerization due to increased chemical interactions between the fragments that enhances the susceptibility of the system. The increased non-linear susceptibility of the materials permits to be used in compact, small, and low power devices for the telecommunication purpose^75^.

##### Non-linear refractive index (n_2_)

The refractive index, ‘n’ can be expressed as n = n_0_ + n_2_ < E^2^ > , where static refractive index n_0_ and non-linear refractive index ‘n_2’_ do not depend on the intensity of light and n_0_ >  > n_2_ and < E^2^ > is the mean square of the applied electric field^76^. According to Ticha and Tichy and Miller’s rule, the non-linear refractive index is related to χ^(3)^ by using the following relation^77^,

12 $$ n_{2} = \frac{{12\pi \chi^{\left( 3 \right)} }}{{n_{0} }} $$

The value of the non-linear refractive index obtained is listed in Table 3. The non-linear refractive index increases with annealing temperature, which is due to the production of defect states by annealing that enhances local polarizabilities^78^. The high values on ‘n_2_^’^ of the annealed Bi_x_In_35-x_Se_65_ films indicate to be potential candidates for non-linear optical applications.

## Conclusion

The analysis from the above study shows the amorphous-crystalline phase transformation at higher annealing temperatures. The new Bi_2_Se_3_ phases were formed as found from XRD and corresponding vibrational mode change as noticed from Raman study. It showed more structural alteration in the case of 450 °C annealed films than 350 °C films. The transmittance decreased whereas the absorption coefficient increased with annealing and doping content. The annealing induced reduction in direct and indirect optical band gap in all films is attributed to the production of surface dangling bonds around the crystallites during phase transformation. This behavior enables these materials to be useful for the absorber layer for photovoltaic applications. The increase in optical density, extinction coefficient, and decrease in skin depth behavior has been observed with an increase in annealing and Bi concentration. The static linear refractive index and high-frequency dielectric constants increased with temperature and doping content. The nonlinear third-order susceptibility and refractive index were increased consequently with annealing temperature. The large non-linear susceptibility of the materials enables them to be used for optical switching, and telecommunication purposes.

## Data Availability

The data that support the findings of this study are available from the corresponding author upon reasonable request.
