# Author Correction: Observation of high nonlinearity in Bi doped Bi_x_In_35-x_Se_65_ thin films with annealing

**DOI:** 10.1038/s41598-024-60479-8

**Published:** 2024-04-26

**Authors:** P. Priyadarshini, Subhashree Das, D. Alagarasan, R. Ganesan, S. Varadharajaperumal, Ramakanta Naik

**Affiliations:** 1 Department of Engineering and Material Physics, ICT-IOC, Bhubaneswar, 751013 India; 2 Department of Physics, Indian Institute of Science, Bangalore, 560012 India; 3 Centre for Nano Science and Engineering, Indian Institute of Science, Bangalore, 560012 India

Correction to: *Scientific Reports* 10.1038/s41598-021-01134-4, published online 02 November 2021

The Acknowledgments section in the original version of this Article was incomplete.

“The author Dr. Naik would like to thank the Department of Physics, Indian Institute of Science (IISc.) for Optical, Raman, and FESEM measurements.”

now reads:

“The author Dr. Naik would like to thank the Department of Physics, Indian Institute of Science (IISc.) for Optical, Raman, and FESEM measurements. The usage of MNCF and NNCF facilities supported by Meity, DST and MOE are acknowledged.”

The original Article has been corrected.

